# Intelligent Optimization of Gas-Assisted Electrospinning via LLM-Guided Bayesian Inference

**DOI:** 10.3390/mi17050619

**Published:** 2026-05-18

**Authors:** Jun Zeng, Rongguang Zhang, Weicheng Ou, Xuanzhi Zhang, Shize Huang, Xun Chen, Guojie Xu

**Affiliations:** 1 State Key Laboratory of Precision Electronic Manufacturing Technology and Equipment, Guangdong University of Technology, Guangzhou 510006, China; fungomail@foxmail.com (J.Z.);; 2 Key Laboratory of Intelligent Detection and The IoT in Manufacturing, Ministry of Education, Guangdong University of Technology, Guangzhou 510006, China; 3 Experimental Teaching Department, Guangdong University of Technology, Guangzhou 510006, China; 4 School of Biomedical and Pharmaceutical Sciences, Guangdong University of Technology, Guangzhou 510006, China

**Keywords:** gas-assisted electrospinning, Bayesian optimization, large language model, nanofiber uniformity, semiconductor manufacturing

## Abstract

Nanofiber-based structures have shown considerable potential in semiconductor-related applications, including ultra-thin dielectric layers and flexible electronic devices, owing to their tunable micro-/nanoscale morphology. However, the manufacturing of these structures is often hindered by the complex multiparameter coupling and poor reproducibility inherent in conventional electrospinning processes. To address these challenges, this study develops an intelligent optimization framework for gas-assisted electrospinning by integrating Large Language Models (LLMs) with Bayesian Optimization (BO). A Gaussian Process Regression (GPR) surrogate model was established to navigate the high-dimensional parameter space efficiently. Comparative studies demonstrate that the proposed BO+LLM strategy not only outperforms pure data-driven BO and pure knowledge-driven LLM approaches but also surpasses the conventional Response Surface Methodology (RSM) baseline, successfully locating a verified minimum fiber diameter of 239 nm. Furthermore, through response-surface analysis, this work identifies a specific multiphysics collaborative window where electrostatic stretching and aerodynamic assistance are balanced. These findings provide a robust pathway for the reproducible fabrication of nanofiber-based electronic devices.

## 1. Introduction

Nanofiber materials have attracted increasing interest in semiconductor-related applications because of their high specific surface area, tunable micro-/nanostructures, and tailorable interfacial characteristics, particularly in flexible electronic devices, optoelectronic detection, gas sensing, functional interfaces, and ultra-thin dielectric layers [1,2,3,4,5,6,7,8,9,10]. In such systems, ultrafine fibrous architectures can further regulate interfacial properties, charge-transport pathways, and the spatial distribution of functional layers, making them promising structural units for next-generation nanoelectronic devices and semiconductor functional materials [11,12,13,14]. From this perspective, the practical value of nanofiber fabrication lies not only in achieving small structural dimensions, but also in ensuring controllable morphology, structural consistency, and process reproducibility that are compatible with device manufacturing.

Electrospinning is one of the most important techniques for the continuous preparation of micro- to nanoscale fibers. Owing to its relative simplicity, broad material compatibility, and strong tunability in fiber diameter, morphology, and internal structure, it has been widely used for the preparation of polymeric, inorganic oxide, composite, and functional semiconductor fibers [15,16,17,18,19]. Nevertheless, for semiconductor-device applications, the ability to merely produce fibers is far from sufficient. More critically, the fabrication process must provide stable control over fiber size, diameter distribution, and structural uniformity during continuous production so as to satisfy the requirements of reproducibility and scalable manufacturing. Previous reviews have consistently pointed out that conventional electrospinning still faces major limitations in process scale-up, morphological consistency, and reproducibility, which remain key obstacles to its engineering implementation and manufacturing use [20,21,22].

To overcome these limitations, gas-assisted electrospinning has gradually emerged as an important development direction. By introducing an auxiliary airflow around the nozzle, aerodynamic stretching can act cooperatively with the electric field, thereby enhancing jet elongation, promoting solvent evaporation, and improving both fiber productivity and deposition stability. Previous studies have shown that gas-assisted spinning and electro-blowing approaches offer clear advantages in increasing production efficiency, improving jet behavior, and enabling scalable fabrication, thus providing a promising route for translating electrospun nanofibers toward manufacturing [23,24,25,26]. This feature is particularly attractive for semiconductor-related nanofiber structures, where improved diameter uniformity and structural controllability are essential for dielectric layers, sensing layers, and flexible functional substrates.

However, the introduction of airflow also makes the electrospinning process substantially more complex. Under the coupled effects of airflow, electric field, and solution flow, fiber formation is simultaneously influenced by solution flow rate, solution concentration, applied voltage, air pressure, and nozzle geometry, and these variables often exhibit pronounced nonlinear coupling relationships. Existing studies and reviews have shown that parameter variation affects not only the average fiber diameter, but also the diameter distribution, structural integrity, and the stability range of the process window [20,21,22,23,24,27]. As a result, the key challenge is no longer simply to determine whether a parameter matters, but to identify how multiple parameters interact to create or destroy a favorable processing region. This issue is particularly important for semiconductor applications, because device manufacturing requires a stable and reproducible process window rather than an isolated optimum achieved only once.

Traditionally, electrospinning parameter optimization has relied mainly on empirical experiments or classical design-of-experiment approaches, such as single-factor experiments and response surface methodology (RSM). Although these methods can reveal, to some extent, the relationships between individual process parameters and fiber diameter, they usually require a large number of experiments to generate reliable conclusions in highly coupled multivariable systems. In addition, their ability to capture higher-order nonlinear interactions is limited, making them inefficient for the systematic exploration of complex process spaces [20,21,28]. Therefore, for gas-assisted electrospinning, the central methodological problem is how to achieve efficient, reliable, and interpretable optimization under limited experimental data while still identifying physically meaningful process windows.

In recent years, Bayesian optimization (BO) has attracted widespread attention as an effective approach for optimizing expensive black-box functions in materials design, experimental optimization, and autonomous experimentation systems. BO constructs a probabilistic surrogate model, typically Gaussian process regression (GPR), to describe the relationship between input parameters and target outputs, and uses an acquisition function to balance exploration of unknown regions with exploitation of known favorable regions, thereby iteratively recommending new experimental conditions [29,30]. Compared with conventional approaches that rely on dense experimental sampling, BO can approach the optimum with far fewer experiments, making it especially suitable for process-optimization tasks involving high experimental cost, large parameter spaces, and substantial process noise [29,30,31]. Nevertheless, conventional BO remains essentially data-driven: it depends mainly on statistical learning from existing observations and does not explicitly incorporate mechanistic understanding or expert knowledge. As a consequence, its search efficiency and the rationality of the recommended candidate points may still be limited at the small-sample stage.

At the same time, large language models (LLMs) have shown increasingly strong capabilities in knowledge integration, textual reasoning, scientific information extraction, and research assistance [32,33,34]. Recent studies have indicated that LLMs can not only extract structured experimental information from the scientific literature, but can also contribute to scientific problem analysis, experimental design, and knowledge-guided candidate generation in materials-research workflows. Based on these studies, we formulate the working hypothesis that LLMs may provide a form of domain-informed prior knowledge beyond the available experimental dataset. In the present study, this hypothesis refers specifically to the possibility that an LLM can propose more feasible and better-directed candidate parameter combinations by integrating literature trends, physical intuition, and historical experimental observations. When combined with BO, such LLM-assisted candidate generation is expected to complement probabilistic search: the language model provides physically meaningful suggestions, whereas BO continuously updates the surrogate model and refines the search direction through experimental feedback. The validity of this hypothesis is evaluated in this work through comparative optimization experiments.

Based on this working hypothesis, this study proposes an intelligent optimization framework that combines LLM-assisted knowledge guidance with BO-based iterative search for gas-assisted electrospinning parameter optimization. First, a GPR-based surrogate model is constructed from existing experimental data to establish the predictive relationship between process parameters and fiber diameter. Then, Bayesian optimization iteratively explores the process space, while the LLM provides domain-informed candidate parameters to improve search efficiency and exploratory capability. Through this collaborative strategy, favorable regions for fiber refinement and improved uniformity can be identified more efficiently under a limited number of experiments, and the coupling mechanisms among key process parameters can be further clarified. In addition, a retrospective RSM baseline is constructed using selected historical experimental samples to provide a conventional optimization reference and to compare the reliability of one-shot polynomial prediction with adaptive experimental-feedback-driven optimization. More importantly, the goal of this work is not only to reduce fiber diameter itself, but also to address the broader manufacturing problem of uniformity, reproducibility, and scalable regulation in semiconductor-related nanofiber fabrication, thereby providing a feasible intelligent process-optimization route for extending electrospun nanofibers toward semiconductor functional layers and device manufacturing.

## 2. Materials and Methods

### 2.1. Materials

The polymer used in this study was polyvinyl alcohol (PVA-220, Kuraray, Tokyo, Japan), supplied by Aladdin Chemical Co. (Shanghai, China). All reagents were of analytical grade and were used without further purification. PVA aqueous precursor solutions with polymer concentrations of 10–16 wt% were prepared by dissolving a calculated amount of PVA powder in deionized water under continuous stirring until a homogeneous and transparent solution was obtained. The solution was allowed to stand before electrospinning to remove visible bubbles and was then loaded into a syringe for subsequent gas-assisted electrospinning experiments. The polymer concentration was adjusted according to the experimental design and was used as one of the input variables for model construction and process optimization.

### 2.2. Gas-Assisted Electrospinning Device and Experiment

**Figure 1:**
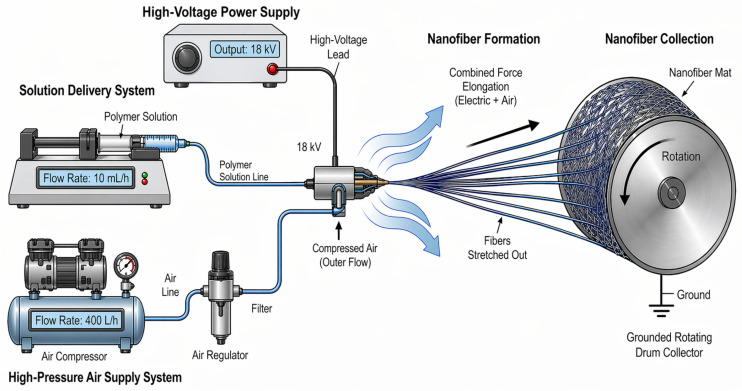
Schematic diagram of the gas-assisted electrospinning setup.

A self-built gas-Assisted Electrospinning system was employed in this work, and its schematic is shown in Figure 1. The system mainly consisted of a high-voltage power supply (J2, DongWen High Voltage Power Supply (TianJin) Co., Ltd.), a syringe pump (LSP01-1A, Baoding Longer Pump Co., Ltd.), a compressed-air system (BD-7.5EPM Guangdong Baldor Technology Co., Ltd.), a gas-assisted nozzle (Foshan Wemaxnano Technology Co., Ltd.), and a grounded rotating drum collector (Foshan Wemaxnano Technology Co., Ltd.). During the experiment, the PVA polymer solution was delivered to a metal needle at a preset flow rate by the syringe pump through a transfer tube. A high-voltage electric field was applied to the needle through a high-voltage cable. Meanwhile, the compressed-air system provided a high-pressure airflow which, after regulation and filtration, entered the annular gas channel surrounding the nozzle. Near the nozzle outlet, the high-speed airflow and the electric field acted jointly on the jet. A Taylor-cone droplet formed at the needle tip was stretched and accelerated toward the grounded rotating drum collector.

The specific ranges for these processing parameters are summarized in Table 1. All experiments were conducted under controlled environmental conditions at a temperature of 27 ± 1 °C and a relative humidity of 45 ± 3% RH. To establish the initial dataset for model training, an experimental design was performed within the ranges specified in Table 1. Specifically, 40 distinct experimental trials (N = 40) were conducted, with parameters varied in the following intervals: polymer concentration *C* at 2 wt% increments, *V* at 2 kV increments, solution flow rate *Q* at 0.5 mL/h increments, *D* at 3 cm increments, $P_{air}$ at 0.1 MPa increments, and $L_{needle}$ at 5 mm increments. To ensure consistency, each experiment was repeated three times, and fibrous membranes were collected after a deposition time sufficient to guarantee a stable sample weight (>0.5 g) for subsequent characterization. These 40 sets of data were then used to train the GPR model, with subsets of 20, 30, and 40 data points extracted in sequence to evaluate the model’s performance at different sample sizes.

### 2.3. Morphological Characterization of Nanofibers

The prepared nanofiber membranes were characterized by scanning electron microscopy (SEM) to observe their morphology. The SEM images were used to analyze fiber surface structure and the uniformity of fiber distribution.

For each experimental condition, three independently fabricated membranes were prepared. For each membrane, 50 fiber-diameter measurements were randomly collected from SEM images using ImageJ 2.17.0. The average fiber diameter and standard deviation were calculated from these measurements to obtain statistically meaningful diameter distributions. The final average fiber diameter of each condition was used as the response variable for GPR model training and optimization.

### 2.4. Construction of the GPR Surrogate Model and Kernel Selection

To establish a predictable mapping between gas-assisted electrospinning process parameters and fiber diameter, Gaussian process regression (GPR) was adopted as the data-driven surrogate model. The model took six key process variables as input features, including polymer concentration *C*, applied voltage *V*, solution flow rate *Q*, emitter–collector distance *D*, air pressure $P_{air}$, and needle extension $L_{needle}$, while the output was the experimentally measured average fiber diameter. Considering that the kernel function plays a decisive role in the fitting capability and generalization performance of the GPR model, three commonly used kernels were systematically evaluated: the radial basis function (RBF) kernel, the Matern 3/2 kernel, and the Matern 5/2 kernel. To reduce the sensitivity of model performance to the train/test split, Model performance was evaluated under different total sample sizes using a unified 7:3 training/validation split, and the robustness of the selected setting was further examined by LOOCV. The robustness of the model was comprehensively evaluated using R^2^, root mean square error (RMSE), and mean absolute error (MAE), and the optimal data-partition strategy was selected accordingly. Under this unified strategy, the candidate kernels were fairly compared, and, together with an agreement analysis based on predicted-versus-measured scatter plots, the kernel with the best fitting accuracy and extrapolation capability was ultimately selected as the default surrogate model for subsequent optimization.

### 2.5. BO-Driven Parameter Recommendation Mechanism

Based on the GPR surrogate model, Bayesian optimization (BO) was used to iteratively minimize fiber diameter. In each optimization round, a large number of candidate points were generated within the predefined physically feasible parameter space, and the current GPR model was used to predict the mean fiber diameter and uncertainty at each point. Subsequently, the expected improvement (EI) acquisition function was used to evaluate the potential gain of each candidate point relative to the current best result. The EI function naturally balances exploitation (fine search in low-prediction-value regions) and exploration (trying new parameters in regions of high uncertainty), thereby effectively avoiding entrapment in local minima. Under the present setting, the BO module output three high-potential candidate parameter combinations in each iteration as data-driven experimental recommendations for the next round of gas-assisted electrospinning experiments.

### 2.6. LLM-Based Knowledge Guidance and the BO+LLM Collaborative Optimization Loop

To compensate for the limited exploratory efficiency of purely data-driven methods at the small-sample stage and to improve the rationality of the search through domain knowledge, a large language model (LLM) was further incorporated as a knowledge-guided parameter generator operating in parallel with Bayesian optimization. At the beginning of each iteration, the LLM received a structured prompt including a summary of the historical experimental results, particularly the well-performing parameter–diameter combinations, the allowable ranges of all process variables, the explicit optimization objective of minimizing fiber diameter, and the necessary engineering feasibility constraints. Based on this contextual information, the LLM generated two candidate parameter sets that were consistent with physical intuition and process knowledge. These candidates were then merged with the three candidates proposed by Bayesian optimization, followed by deduplication and boundary checking, to form the experimental plan for the next iteration. After the experiments were completed, the newly obtained fiber-diameter data were added to the dataset and used to update the surrogate model.

**Figure 2:**
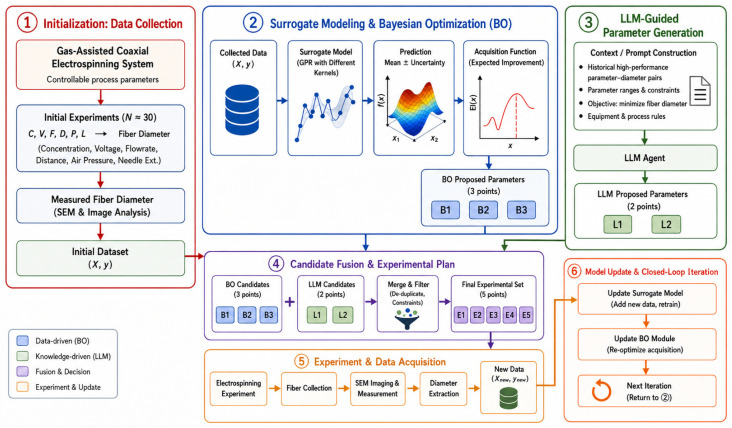
Overall framework of the collaborative LLM- and BO-driven parameter optimization workflow.

For clarity, the overall collaborative optimization workflow is illustrated in Figure 2. The framework combines a data-driven search branch with a knowledge-guided candidate-generation branch and forms a closed-loop iterative process through candidate fusion and experimental feedback. In this process, Bayesian optimization performs probabilistic search in the parameter space on the basis of the surrogate model, whereas the LLM proposes physically plausible candidates by using historical experimental information and process constraints. After experimental evaluation, the resulting data are continuously fed back into the surrogate model, enabling efficient exploration of the process-parameter space.

In each iteration, the LLM generated two candidate parameter sets. Each suggested candidate was required to include the complete values of polymer concentration, applied voltage, solution flow rate, emitter–collector distance, air pressure, and needle extension, together with a short explanation of the expected effect on fiber refinement. The LLM-generated candidates were then merged with three candidates proposed by Bayesian optimization. After deduplication, boundary checking, and feasibility screening, the final candidate set was used for the next round of gas-assisted electrospinning experiments. After the experiments were completed, the newly measured fiber-diameter data were added to the dataset and used to update the GPR surrogate model for the next iteration.

For clarity, the overall collaborative optimization workflow is illustrated in Figure 2. The BO branch performs probabilistic search based on the GPR surrogate model and the expected improvement acquisition function, while the LLM branch provides knowledge-guided candidate suggestions based on historical experimental trends and physical process constraints. Through candidate fusion and experimental feedback, the BO+LLM framework forms a closed-loop optimization process in which statistical learning and domain-informed reasoning are combined to explore the process-parameter space more efficiently.

### 2.7. Retrospective RSM Baseline and Validation Design

To provide a conventional design-of-experiments-based reference, a retrospective RSM baseline was constructed following the principle of D-optimal response surface design. Based on the predefined process ranges and experimental feasibility constraints of the gas-assisted electrospinning system, 30 representative experimental conditions were organized to cover the main parameter space, including boundary conditions, low-diameter regions, and interior support points. This design was used to fit a reduced quadratic RSM model containing main effects, quadratic effects, and selected pressure-related interaction effects. The input variables included including polymer concentration *C*, applied voltage *V*, solution flow rate *Q*, emitter–collector distance *D*, air pressure $P_{air}$, and needle extension $L_{needle}$, while the response variable was the measured average fiber diameter.

## 3. Results and Discussion

### 3.1. Sample-Size- and Kernel-Dependent GPR Model Selection with Robustness Validation

**Figure 3:**
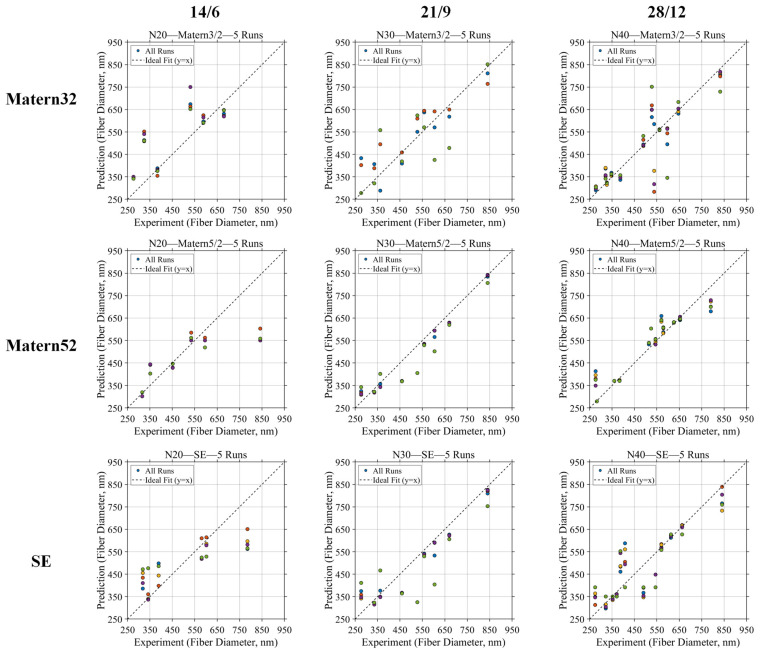
Predicted-versus-experimental fiber diameter scatter plots under different kernel functions and sample sizes. Data points (circles) sharing the same color indicate the results obtained during a single iteration cycle.

To identify a surrogate model that could provide both sufficient predictive reliability and acceptable experimental economy, the GPR model was evaluated from two coupled aspects: the available sample size and the kernel function. As shown in Figure 3, three sample sizes, N = 20, N = 30, and N = 40, were compared under the same 7:3 training/validation strategy, corresponding to 14/6, 21/9, and 28/12 training/validation samples, respectively. For each sample size, three kernel functions, namely Matern 3/2, Matern 5/2, and squared exponential (SE), were tested. The quantitative validation results are summarized in Table 1. The purpose of this comparison was not simply to obtain the lowest error with the largest dataset, but to determine the earliest sample scale at which the six-dimensional process–diameter relationship could be described with sufficient stability for subsequent BO-based optimization.

As shown in Figure 3 and Table 2, the predictive behavior changed significantly with increasing sample size. At N = 20, the validation points of all three kernels showed noticeable scattering relative to the ideal y = x line. Although the three kernels produced different local distributions, none of them provided a stable and convincing prediction pattern. This is also reflected by the relatively low R^2^ values of 0.44–0.56 and RMSE values above 100 nm in Table 2. Therefore, N = 20 was considered insufficient for representing the present gas-assisted electrospinning parameter space, especially considering that six process variables were involved. In other words, at this stage the model was still strongly constrained by the sparsity of the available samples, and the apparent fitting result could not be regarded as a reliable basis for guiding further optimization.

When the sample size increased to N = 30, a clear improvement appeared. The prediction points became more concentrated around the ideal line, and the difference among kernel functions became more meaningful. As summarized in Table 2, the Matern 5/2 model achieved the best overall performance at this sample size, with R^2^ = 0.88, RMSE = 54 nm, and MAE = 40 nm. Compared with Matern 3/2 and SE at the same N = 30 condition, the Matern 5/2 model showed a better ability to follow the overall measured–predicted relationship rather than only fitting a limited local range. This suggests that the response surface of fiber diameter in the present process is not completely smooth or strictly linear, but contains moderate nonlinear variations caused by the coupled effects of *V*, *Q*, *D*, $P_{air}$, *C*, and $L_{needle}$. Under such conditions, Matern 5/2 provides a more suitable balance between smoothness and flexibility than the other tested kernels.

Further increasing the sample size to N = 40 improved the general availability of training information, and all three kernels showed acceptable prediction capability. According to Table 2, the SE kernel gave the lowest RMSE and MAE values at N = 40, while Matern 5/2 still maintained comparable predictive performance with R^2^ = 0.84, RMSE = 64 nm, and MAE = 39 nm. However, the change from N = 30 to N = 40 should be interpreted as an incremental refinement rather than a qualitative transition in model reliability. Compared with the obvious improvement from N = 20 to N = 30, the additional benefit of N = 40 was obtained at the cost of ten more experimental samples. Since the subsequent optimization framework aims to operate under limited experimental budgets, N = 30 was therefore identified as the minimum practically usable sample size, and Matern 5/2 was selected as the most appropriate kernel function for constructing the initial GPR surrogate model.

To further verify that the selected N = 30 setting was not merely a favorable result caused by one specific 7:3 split, LOOCV was performed for the Matern 5/2 models at N = 30 and N = 40, as shown in Figure 2. In this validation, each sample was removed once as an independent validation point, while the remaining N−1 samples were used to train the model. Therefore, LOOCV provides a stricter assessment of small-sample robustness than a single hold-out split. The corresponding LOOCV metrics are summarized in Table 2. For N = 30, the LOOCV result achieved R^2^ = 0.83, RMSE = 55 nm, and MAE = 35 nm, and most prediction points remained distributed near the ideal line. This confirms that the N = 30 Matern 5/2 model retained acceptable generalization ability even when each sample was independently excluded from training.

**Figure 4:**
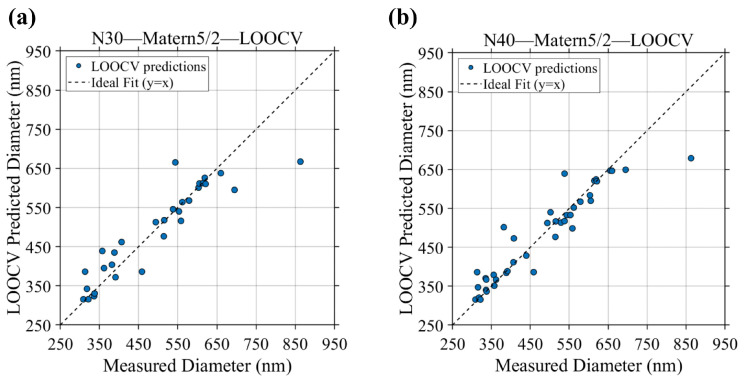
Measured-versus-predicted fiber diameter results obtained by leave-one-out cross-validation for the Matern 5/2 GPR model: (**a**) N = 30; (**b**) N = 40.

When N was increased to 40, the LOOCV performance further improved, with R^2^ = 0.87, RMSE = 47 nm, and MAE = 29 nm, as shown in Table 3. This indicates that additional samples can indeed refine prediction accuracy. However, the improvement from N = 30 to N = 40 was moderate rather than decisive, supporting the conclusion that N = 30 already provides a robust lower-bound sample size for initiating the optimization workflow, while N = 40 mainly enhances model accuracy further. Taken together, the hold-out validation results in Figure 3 and Table 2, together with the LOOCV results in Figure 4 and Table 3, demonstrate that N = 30 with Matern 5/2 represents the minimum reliable surrogate-model setting, whereas N = 40 provides additional but non-essential refinement. Therefore, the N = 30 Matern 5/2 GPR model was adopted as the surrogate basis for the following BO and BO+LLM optimization.

### 3.2. Comparative Iterative Performance of Different Optimization Strategies

**Figure 5:**
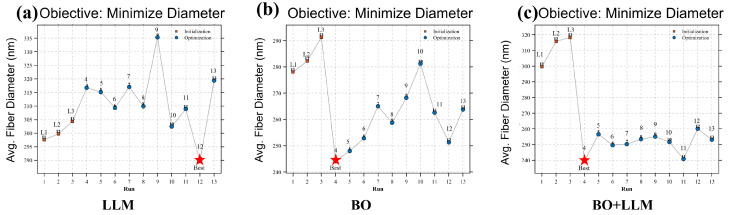
Comparison of iterative optimization trajectories for fiber-diameter minimization under different strategies: (**a**) LLM; (**b**) BO; (**c**) BO + LLM.

After determining the Matern 5/2 kernel as the most suitable surrogate setting and adopting N = 30 with a 7:3 split as the unified data-partition strategy, the iterative search behaviors of the three optimization approaches were further compared on the basis of real experimental feedback. As shown in Figure 5, the three methods exhibited clearly different search trajectories once the optimization stage began. To further quantify the iterative performance, the best experimentally verified diameter, best run, optimization-stage average diameter, and low-diameter retention rate were calculated. The optimization stage refers to Runs 4–13 after the three initialization runs. For engineering-oriented comparison, the low-diameter region was defined as *d* ≤ 260 nm. This threshold was chosen because, in the context of gas-assisted PVA electrospinning, a diameter of 260 nm represents a relatively fine fiber-diameter level within the experimentally achievable range of this process. The low-diameter retention rate was then calculated as the proportion of optimization-stage runs remaining within this threshold.

The LLM-only approach, shown in Figure 5a, displayed the weakest convergence behavior and the strongest fluctuation. During the initialization stage, the fiber diameter increased gradually rather than decreased, indicating that no effective convergence was established at the beginning. In the subsequent optimization stage, the results remained scattered within a relatively broad range, and although the best value of this method was eventually reached at a later iteration, it was not maintained thereafter. Quantitatively, the best LLM-only result was obtained at Round 12, with an average fiber diameter of 289 nm. Its optimization-stage average diameter was 312.39 nm, and none of the optimization-stage runs entered the low-diameter region of *d* ≤ 260 nm. This behavior suggests that the LLM-only approach functioned mainly as a heuristic candidate generator based on contextual knowledge, rather than as a stable optimization process progressively corrected by experimental feedback.

The BO-only approach, shown in Figure 5b, exhibited stronger early search capability but limited stability after reaching a favorable region. After several relatively high initial values, a sharp decrease appeared at the beginning of the optimization stage, and the best value of this method was obtained as early as Round 4, with an average fiber diameter of 244 nm. However, the subsequent iterations did not continue to improve around that optimum; instead, the results rebounded and fluctuated over a wider interval. This indicates that Bayesian optimization alone was able to identify a promising parameter region at an early stage, but its ability to remain stably within that low-diameter region was still limited under the present small-sample conditions. Although BO entered the low-diameter region early, its subsequent rebound increased the optimization-stage average diameter to 259 nm and reduced the low-diameter retention rate to 50%.

By comparison, the collaborative strategy shown in Figure 5c exhibited the most favorable overall search behavior. Similar to the BO-only method, it also achieved a sharp decrease at an early stage and reached the lowest experimentally verified diameter of 239 nm at Round 4. It maintained a lower optimization-stage average diameter of 251 nm and achieved the highest low-diameter retention rate of 90%, indicating better retention of the favorable parameter region.

As summarized in Table 4, BO+LLM achieved the lowest best diameter, the lowest optimization-stage average diameter, and the highest low-diameter retention rate, demonstrating better convergence and retention of favorable parameter regions than the two single-strategy baselines. To further evaluate its advantage against a conventional design-of-experiments-based optimization approach, a retrospective RSM baseline was established and analyzed in the following section.

### 3.3. Retrospective RSM Baseline and Comparison with Adaptive Optimization Strategies

To provide a conventional optimization reference, a retrospective response surface methodology (RSM) baseline was constructed using 30 selected experimental samples. Different from BO-based optimization strategies, RSM is not an adaptive sequential optimizer. It first fits a polynomial response surface from a predefined experimental dataset and then predicts an optimum within the selected parameter bounds. Therefore, in this study, RSM was used as a retrospective baseline rather than as an iterative optimization trajectory.

The average fiber diameter *d* was modeled as a function of six coded process variables, including *C*, *V*, *Q*, *D*, $P_{air}$, and $L_{needle}$. A general second-order RSM model can be expressed as

(1) $$d=\beta_{0}+\sum_{i}{\beta_{i}x}_{i}+\sum_{i}\beta_{ii}x_{i}^{2}+\sum_{i<j}\beta_{ij}x_{i}x_{j}$$

where $x_{i}$ represents the coded process variable and *d* is the measured average fiber diameter. Considering the limited sample size and the important role of air pressure in gas-assisted electrospinning, a reduced quadratic RSM model was adopted instead of a full second-order polynomial. The model included six linear terms, six quadratic terms, four pressure-related interaction terms, and one intercept term. The fitted RSM model is expressed as follows:

(2) $${\hat{d}}_{RSM}=699.87+154.53x_{1}-81.83x_{2}+66.05x_{3}-22.04x_{4}-51.59x_{5}+10.50x_{6}\;+45.29x_{1}^{2}+143.75x_{2}^{2}-261.81x_{3}^{2}-111.10x_{4}^{2}+21.29x_{5}^{2}+158.33x_{6}^{2}\;+10.82x_{1}x_{5}+4.44x_{2}x_{5}-3.74x_{3}x_{5}+12.76x_{4}x_{5}$$

where $x_{1}$, $x_{2}$, $x_{3}$, $x_{4}$, $x_{5}$, and $x_{6}$ correspond to the coded values of *C*, *V*, *Q*, *D*, $P_{air}$, and $L_{needle}$, respectively. The selected interaction terms were mainly associated with air pressure, because the preceding parameter-response analysis indicated that air pressure plays a central role in the coupled regulation of fiber diameter.

**Figure 6:**
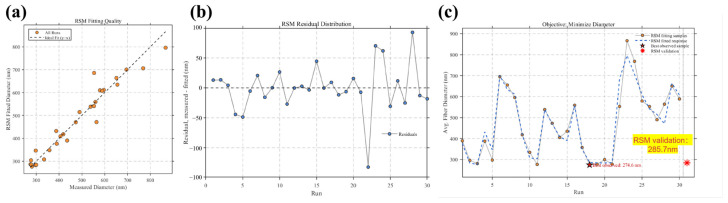
Retrospective RSM baseline evaluation and validation: (**a**) measured-versus-fitted scatter plot of the reduced quadratic RSM model; (**b**) residual distribution of the RSM fitting results; (**c**) RSM fitting samples, fitted response, and experimental validation at the RSM-predicted optimum.

As shown in Figure 6, the 30 selected samples were used to fit the reduced quadratic response surface, and the RSM-predicted optimum was subsequently validated experimentally. The RSM model predicted the minimum fiber diameter at *C* = 10 wt%, *V* = 12.5 kV, *Q* = 0.5 mL/h, *D* = 18 cm, $P_{air}$ = 0.5 MPa, and $L_{needle}$ = 15 mm., with a predicted diameter of 94 nm. However, the experimentally measured average fiber diameter at this predicted optimum was 285 nm. This considerable deviation indicates that the RSM model overestimated the improvement near the boundary of the selected design space.

The fitting quality of the RSM model is shown in Figure 6b. The measured-versus-fitted points are generally distributed around the ideal y = x line, and the model achieved R^2^ = 0.93, RMSE = 39 nm, and MAE = 25 nm. These results suggest that the reduced quadratic RSM model can describe the overall trend of the selected 30-sample dataset with reasonable fitting accuracy. More importantly, the validated RSM diameter was still clearly higher than the best BO+LLM result of 239 nm and the best BO-only result of 244 nm. Specifically, the RSM validation result was 46 nm higher than BO+LLM and 41 nm higher than BO, indicating that the RSM baseline was not only affected by prediction deviation, but also showed a clear performance disadvantage compared with the adaptive BO-based strategies.

This result highlights the difference between RSM and adaptive optimization strategies. RSM can provide a useful retrospective baseline However, its experimentally validated optimum of 285 nm was inferior to both BO and BO+LLM, showing that the RSM baseline was less effective in locating the experimentally verified low-diameter region. In contrast, the BO-based strategies update the search direction through iterative experimental results. The LLM-only strategy can generate physically plausible candidates, but its search trajectory fluctuates strongly because it lacks surrogate-model correction. The BO-only strategy can identify a favorable region at an early stage, but its subsequent results show rebound and instability. By comparison, the BO+LLM strategy combines knowledge-guided candidate generation with surrogate-based iterative refinement, allowing it to enter the low-diameter region early and remain there more stably. Therefore, compared with LLM-only, BO-only, and the retrospective RSM baseline, the BO+LLM framework provides a more reliable optimization route for data-limited and strongly coupled gas-assisted electrospinning.

### 3.4. Analysis of Parameter-Response Characteristics and Coupling Relationships

**Figure 7:**
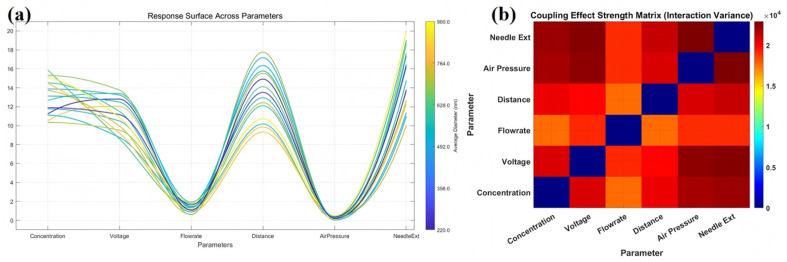
Analysis of parameter-response trends and pairwise coupling effects based on the iteratively updated GPR model: (**a**) response trajectories of average fiber diameter across the six process parameters; (**b**) coupling effect strength matrix of pairwise process.

To further clarify how individual process parameters affect fiber diameter and to identify the parameter pairs that should be prioritized in subsequent detailed experiments, the response trajectories of the six input variables and their pairwise coupling strengths were analyzed on the basis of the iteratively updated GPR model. As shown in Figure 7a, the predicted response curves across the six parameters reveal that the present system does not follow a simple monotonic dependence on every variable. Among the six parameters, flow rate and air pressure exhibit the most pronounced low-value regions, indicating that they play a dominant role in determining whether a favorable fiber-refinement region can emerge. In contrast, *D* and $L_{needle}$ show relatively strong upward trends in the higher-response region, suggesting that excessive jet flight distance or excessive droplet exposure length tends to preserve a larger fiber diameter. By comparison, the response trends along the *V* and *C* axes are less straightforward and do not support a simple one-parameter rule such as “higher voltage always produces finer fibers” or “lower concentration always leads to smaller diameter”. This suggests that the effects of these variables are more strongly governed by their interactions with other process parameters than by independent main effects alone.

This interpretation is consistent with the coupling-strength matrix shown in Figure 7b. The strongest pairwise interactions are observed for $P_{air}$–$L_{needle}$, *V*–$L_{needle}$, and *V*–$P_{air}$, whereas the couplings involving flow rate are comparatively weaker. These results indicate that, although flow rate has a strong influence on fiber diameter, its role is mainly expressed as a dominant main-effect parameter: it directly affects fiber thickness by changing the liquid-feed load, initial jet volume, and solvent-evaporation burden, but does not substantially reshape the action mode of the other variables. In contrast, $P_{air}$, *V*, and $L_{needle}$ form a more strongly coupled regulation group, jointly modifying the local environment near the nozzle exit and thereby changing the jet-evolution pathway.

More specifically, air pressure controls the strength of aerodynamic stretching and the local evaporation environment, voltage determines the electrostatic stretching force imposed on the jet, and needle extension changes both the droplet exposure position and the spatial overlap between the electric field and the airflow. When these parameters vary in combination, jet acceleration, solvent evaporation, and instability development change simultaneously, causing the location and width of the low-diameter region to depend strongly on parameter matching rather than on the extreme value of any single variable. This also explains why voltage does not show the clearest main-effect trend in the response trajectories, yet appears among the strongest interacting variables in the coupling matrix.

This result provides an important basis for the subsequent analysis. The GPR model identifies which parameter pairs are strongly coupled, but the statistical response itself does not fully explain why these couplings occur. In particular, the strong interactions among *V*, $P_{air}$, and $L_{needle}$ imply that the low-diameter region is associated with the balance between electrostatic stretching, airflow-assisted stretching, droplet exposure, and jet stability. Therefore, a force-balance model is introduced in the following section to provide a mechanistic interpretation of the parameter-response behavior observed in Figure 7 and to guide the localized experimental validation and response-surface analysis.

### 3.5. Force-Balance Model for Electrohydrodynamic–Aerodynamic Coupling

**Figure 8:**
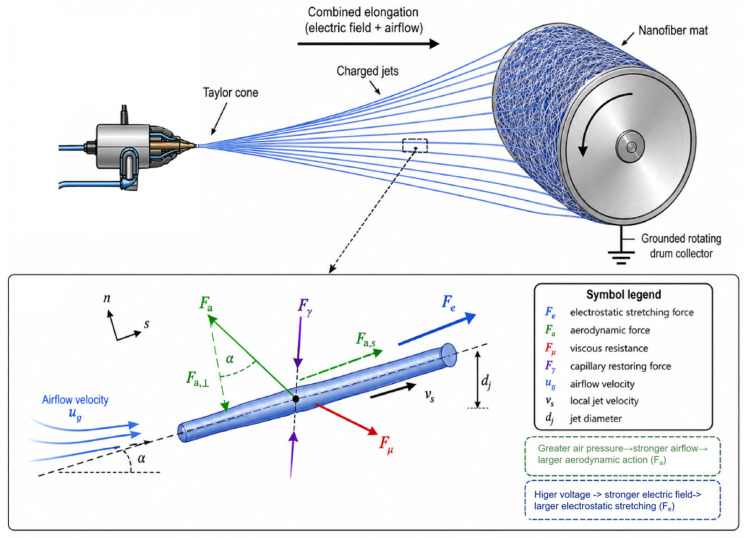
Force-balance model of electrohydrodynamic–aerodynamic coupling in gas-assisted electrospinning.

To provide a physical interpretation for the coupled parameter effects, a simplified force-balance model was established for a representative jet element in the gas-assisted electrospinning process. As shown in Figure 8, the charged jet is stretched from the Taylor cone toward the grounded rotating drum under the combined action of the electric field and auxiliary airflow. A local jet element in the stretched region was selected for force analysis, the local coordinate s is defined along the jet axis, while nnn denotes the transverse direction.

The major forces acting on the jet include the electrostatic stretching force $F_{e}$ aerodynamic force $F_{a}$, viscous resistance $F_{\mu }$, and capillary restoring force $F_{\gamma }$ Along the jet axis, the simplified momentum balance can be written as

(3) $$\rho_{l}A_{j}v_{s}\frac{dv_{s}}{ds}=\lambda_{q}E_{s}+f_{a,s}-f_{\mu ,s}-f_{\gamma ,s}$$

where $\rho_{l}$ is the liquid density, $A_{j}$ is the jet cross-sectional area, $v_{s}$ is the local jet velocity, $\lambda_{q}$ is the charge per unit length, and $E_{s}$ is the electric-field component along the jet axis. The terms $f_{a,s},\;f_{\mu ,s}$ and $f_{\gamma ,s}$ resent the axial aerodynamic force, viscous resistance, and capillary resistance per unit length, respectively. Equation (1) shows that jet thinning is governed by the competition between the stretching terms $\lambda_{q}E_{s}+f_{a,s}$ and the resisting terms $f_{\mu ,s}-f_{\gamma ,s}$.

The applied voltage mainly affects the electrostatic stretching term through the local electric field, which can be approximated as

(4) $$E_{s}∼\eta_{E}\left(L_{needle}\right)\frac{V}{D}$$

where V is the applied voltage, D is the effective collection distance, and $\eta_{E}(L_{needle})$ is a correction factor associated with $L_{needle}$. This term indicates that needle extension changes the local electric-field concentration near the nozzle, and therefore couples with the applied voltage in controlling the initial jet acceleration.

The auxiliary airflow provides an additional aerodynamic action on the jet. The aerodynamic force per unit length can be expressed as

(5) $$f_{a}=\frac{1}{2}C_{D}\rho_{g}d_{j}\left|u_{g}-v_{s}\right|\left(u_{g}-v_{s}\right)$$

where $C_{D}$ is the drag coefficient, $\rho_{g}$ is the gas density, $d_{j}$ is the instantaneous jet diameter, and $u_{g}$ is the airflow velocity. Since the airflow is driven by compressed air,

(6) $$u_{g}\propto \sqrt{\frac{2\left(P_{air}-P_{0}\right)}{\rho_{g}}}$$

where $P_{air}$ and $P_{0}$ are the supplied air pressure and ambient pressure, respectively. Thus, air pressure affects fiber formation by changing the aerodynamic force acting on the moving jet.

As shown in Figure 8, the aerodynamic force can be decomposed into an axial stretching component and a transverse perturbation component:

(7) $$f_{a,s}=\chi \left(L_{needle}\right)f_{a}cos\alpha \;f_{a,⊥}=\chi \left(L_{needle}\right)f_{a}sin\alpha$$

where α is the angle between the airflow direction and the jet axis, and $\chi (L_{needle})$ is the airflow–jet overlap coefficient. A suitable $L_{needle}$ increases the effective interaction between the airflow and the exposed jet, thereby enhancing $f_{a,s}$. However, excessive exposure may increase $f_{a,⊥}$, leading to stronger transverse perturbation and jet instability.

The transverse force balance can be expressed in a simplified form as

(8) $$\rho_{l}A_{j}{v_{s}}^{2}\kappa =f_{a,⊥}+f_{e,⊥}+\delta_{0}-f_{\mu ,⊥}-f_{\gamma ,⊥}$$

where κ is the local curvature of the jet, $f_{e,⊥}$ is the transverse electrostatic perturbation, and $\delta_{0}$ represents initial disturbance. This equation shows that the airflow has a dual effect: the axial component promotes fiber thinning, while the transverse component may increase bending instability.

The relationship between stretching and fiber diameter can be described by mass conservation:

(9) $$Q=A_{j}v_{s}=\frac{\pi {d_{j}}^{2}}{4}v_{s}$$

Therefore

(10) $$d_{f}\propto {\left(\frac{QC_{s}}{v_{s}\left(L\right)}\right)}^{\frac{1}{2}}$$

where Q is the solution flow rate, $C_{s}$ is the effective solid content, and $v_{s}(L)$ is the jet velocity near the collector. This explains why flow rate mainly behaves as a dominant main-effect parameter: increasing Q directly increases the material throughput that must be stretched.

To summarize the competition between stretching and instability, two indicators are introduced:

(11) $$S=\frac{\lambda_{q}E_{s}+f_{a,s}}{f_{\mu ,s}+f_{\gamma ,s}}$$

(12) $$I=\frac{f_{a,⊥}+f_{e,⊥}+\delta_{0}}{T_{s}+f_{\mu ,⊥}+f_{\gamma ,⊥}}$$

where S represents the effective stretching intensity, while I represents the tendency toward transverse instability. A favorable fiber-refinement condition requires a sufficiently high S and a controlled I. This model explains why the low-diameter region is not achieved by simply maximizing voltage or air pressure. Instead, it is formed only when electrostatic stretching and aerodynamic assistance are properly balanced while transverse perturbation remains limited.

Based on this force-balance model, the strong interactions observed in Figure 7b can be interpreted more clearly. The voltage–air pressure pair directly controls the balance between $\lambda_{q}E_{s}$ and $f_{a,s}$. The air pressure–needle extension pair determines how effectively the airflow acts on the exposed jet through $f_{a,s}$, $f_{a,⊥}$, and $\chi (L_{needle})$. The voltage–needle extension pair reflects the combined influence of electric-field concentration and nozzle exposure geometry through $\eta_{E}(L_{needle})$. Therefore, *V*, $P_{air}$, and $L_{needle}$ jointly define the electrohydrodynamic–aerodynamic coupling window for fiber refinement.

### 3.6. Localized Experimental Validation of the Voltage–Air Pressure Coupling

**Figure 9:**
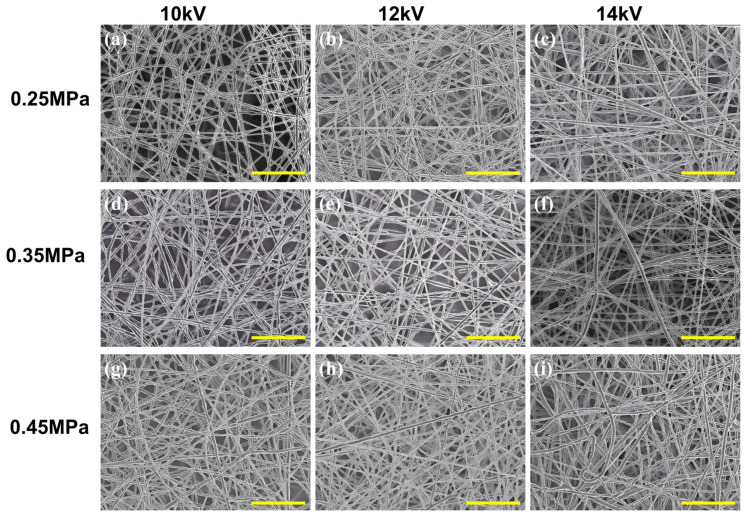
SEM morphology of nanofibers under the voltage–air pressure matrix: (**a**–**c**) 0.25 MPa with 10, 12, and 14 kV; (**d**–**f**) 0.35 MPa with 10, 12, and 14 kV; (**g**–**i**) 0.45 MPa with 10, 12, and 14 kV.

**Figure 10:**
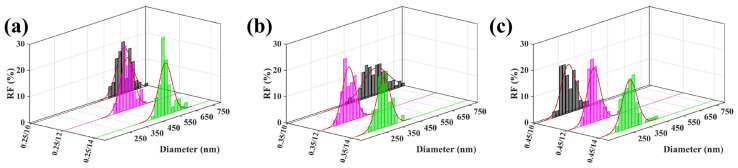
Fiber-diameter distributions under the voltage–air pressure matrix: (**a**) 0.25 MPa; (**b**) 0.35 MPa; (**c**) 0.45 MPa. Each panel compares the distributions at 10, 12, and 14 kV.

Based on the preceding analysis of parameter-response trends and pairwise coupling strengths, the *V*–$P_{air}$ pair was selected for localized experimental validation as one of the most representative strongly coupled parameter combinations. This selection is also consistent with the force-balance model established in Section 3.5, in which *V* mainly determines the electrostatic stretching term *λ_q_E_s_*, whereas air pressure regulates the aerodynamic contribution *f_a_*_,*s*_ and $f_{a,⊥}$ through the airflow velocity $u_{g}$. Therefore, the voltage–air pressure pair directly controls the balance between the stretching index *S* and the instability index I. To verify whether the favorable low-diameter region predicted by the GPR model and interpreted by the force-balance model could be reproduced experimentally, a 3 × 3 parameter matrix was designed under fixed concentration, *Q*, *D*, and $L_{needle}$, while only *V* and $P_{air}$ were varied. As shown in Figure 9a–i, the rows correspond to air pressures of 0.25, 0.35, and 0.45 MPa, and the columns correspond to voltages of 10, 12, and 14 kV. The associated diameter distributions are summarized in Figure 10a–c for the three pressure levels.

A clear trend can be observed along the voltage direction. At each air pressure level, the fibers obtained at 12 kV are generally finer and more uniform than those produced at 10 kV and 14 kV. This result confirms the conclusion of the previous response-surface analysis that the effect of voltage on fiber refinement is not monotonic, but instead exhibits an optimum interval. From the perspective of the force-balance model, when the voltage is too low, the electrostatic stretching term $\lambda_{q}E_{s}$ is insufficient, resulting in a relatively low stretching index *S*. Under this condition, even if auxiliary airflow is introduced, the aerodynamic component cannot be efficiently converted into effective jet elongation, and the refinement effect remains limited. When the voltage is increased to 12 kV, the electric-field-driven stretching becomes sufficiently strong to cooperate with the airflow-assisted stretching, leading to a higher *S* while maintaining a controlled instability level. However, when the voltage is further increased to 14 kV, the jet becomes more susceptible to electrical perturbation and bending disturbance. In this case, the instability index I increases, which weakens the refinement effect and broadens the diameter distribution. Therefore, the role of voltage in the present system is not simply to intensify stretching, but to establish a suitable electrohydrodynamic state under which aerodynamic assistance can act most effectively.

A similarly coupled trend is observed along the air pressure direction. In general, increasing air pressure promotes fiber refinement, but the extent of this improvement depends strongly on the applied voltage. This effect is most evident at 12 kV, where the morphology becomes progressively finer from the first row to the third row, and the smallest average fiber diameter is obtained at 12 kV/0.45 MPa. According to Equations (3)–(6), increasing air pressure raises the airflow velocity $u_{g}$, thereby enhancing the aerodynamic force $f_{a,s}$. When the electrostatic stretching state is appropriate, as in the 12 kV condition, a larger portion of this aerodynamic force can contribute to the axial stretching component $f_{a,s}$, promoting jet elongation and fiber thinning. By contrast, under 10 kV, increasing air pressure leads only to limited refinement because the electrostatic driving force remains insufficient and the overall stretching index is still low. Under 14 kV, the improvement is less stable, indicating that excessive voltage increases the sensitivity of the jet to transverse perturbation; in this case, the beneficial contribution of $f_{a,s}$ is partly counteracted by the increased instability associated with $f_{a,⊥}$, and electric-field disturbance. In this sense, air pressure acts as an aerodynamic amplification factor, but its contribution can only be fully realized under an appropriate electrical condition.

The diameter-distribution results in Figure 10a–c further support this interpretation. At each pressure level, the 12 kV condition generally exhibits a more concentrated and left-shifted distribution than the corresponding 10 kV and 14 kV cases, indicating not only reduced average diameter but also improved process stability and structural uniformity. More importantly, the same relative ranking among the three voltage levels is maintained across different pressure rows, showing that the observed trend is not an isolated fluctuation, but a stable manifestation of the voltage–air pressure coupling relationship.

Taken together, these localized experiments provide direct experimental support for the preceding coupling analysis and response-surface prediction. They show that the favorable low-diameter region is not generated by the extreme value of either voltage or air pressure alone, but by their coordinated matching. The best-performing condition, 12 kV/0.45 MPa, therefore represents not merely a local optimum within the experimental matrix, but the practical expression of a broader multiphysics collaborative window. In this window, electrostatic stretching, aerodynamic assistance, solvent evaporation, and jet stability are balanced simultaneously, corresponding to a sufficiently high stretching index S and a controlled instability index I. This also explains why the favorable region predicted by the model appears as a process window rather than as an isolated optimum point.

### 3.7. Response-Surface Analysis and Summary of the Process Window

**Figure 11:**
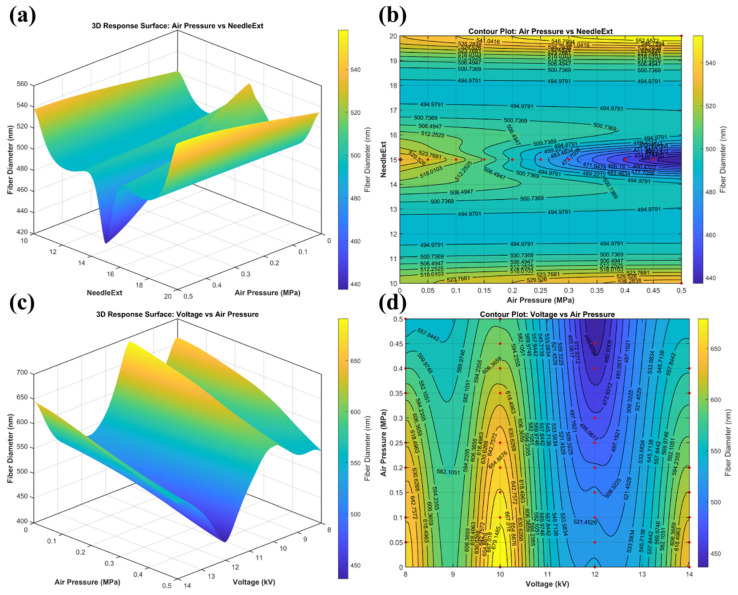
Response-surface analysis of representative coupled process parameters and the resulting low-diameter regions: (**a**,**b**) air pressure–needle extension; (**c**,**d**) voltage–air pressure. The red dots superimposed on the contour plots represent the original experimental data points used for model training.

To further clarify, from the perspective of the continuous parameter space, how the strongly coupled parameter pairs regulate fiber diameter, response-surface analysis was performed for the two representative coupled pairs, namely air pressure–needle extension and voltage–air pressure, based on the updated Matern 5/2 GPR model. The red dots shown in the contour plots (Figure 11b,d) correspond to the actual experimental data points distributed within the parameter space, demonstrating the data density used for the surface fitting. These two parameter pairs were selected because they correspond directly to the key terms in the force-balance model. The air pressure–needle extension pair mainly affects the airflow–jet interaction through the aerodynamic force $f_{a}$, the airflow–jet overlap coefficient $\chi (L_{needle})$, and the decomposition of $f_{a}$ into axial stretching and transverse perturbation components. The voltage–air pressure pair directly controls the balance between electrostatic stretching $\lambda_{q}E_{s}$ and aerodynamic stretching $f_{a}$, which determines whether the jet can reach a favorable fiber-refinement state. For the air pressure–needle extension pair, the remaining parameters were fixed at *C* = 14 wt%, *V* = 12 kV, *Q* = 0.5 mL/h, and *D* = 15 cm. For the voltage–air pressure pair, the fixed values were *C* = 14 wt%, *Q* = 0.5 mL/h, *D* = 15 cm, and $L_{needle}$ = 15 mm.

As shown in Figure 11a,b, the air pressure–needle extension response surface exhibits a clear local valley, indicating that the favorable low-diameter region is concentrated within a limited range rather than extending monotonically with either parameter alone. In particular, the lowest-response region is centered around moderate needle extension and relatively high air pressure, suggesting that fiber refinement depends on the coordinated matching between the droplet exposure position and the aerodynamic stretching intensity. From the force-balance model, increasing air pressure raises the airflow velocity $u_{g}$, thereby increasing the aerodynamic force $f_{a}$. However, whether this aerodynamic force contributes mainly to useful axial stretching $f_{a,s}$ or to transverse disturbance $f_{a,⊥}$, depends strongly on the needle extension. A moderate needle extension provides an effective airflow–jet overlap $\chi (L_{needle})$, allowing the airflow to enhance axial elongation while keeping transverse perturbation controlled. When the needle extension deviates from this intermediate range, the predicted fiber diameter increases again, indicating that either insufficient or excessive droplet protrusion weakens the beneficial overlap between the electric field and the airflow. This result shows that air pressure can effectively promote refinement only when the nozzle geometry provides a suitable exposure condition for stable jet stretching and solvent evaporation.

A similarly coupled feature is observed for the voltage–air pressure pair in Figure 11c,d. The response surface does not show a simple monotonic decrease with increasing voltage or air pressure, but instead displays a relatively broad low-value band located at moderate voltage and relatively high air pressure. This trend is consistent with the stretching–instability balance described in Section 3.5. When the voltage is too low, the electrostatic stretching term $\lambda_{q}E_{s}$ is insufficient, resulting in a low stretching index S. Under this condition, even if air pressure is increased, the airflow-assisted refinement effect cannot be fully utilized because the jet lacks sufficient electrohydrodynamic acceleration. When the voltage is increased to a moderate level, the electrostatic force and the axial aerodynamic component $f_{a,s}$ act cooperatively, leading to effective jet thinning. However, when the voltage is too high, the jet becomes more prone to electrical perturbation and bending instability, increasing the instability index I. As a result, the favorable region appears only when electrostatic stretching and aerodynamic assistance are properly matched.

Taken together, these two response surfaces indicate that the favorable low-diameter region in the present system is not produced by the extreme value of any single parameter, but by the coordinated interaction of electric-field action, aerodynamic stretching, solvent evaporation, and nozzle geometry. In the air pressure–needle extension pair, the key issue is whether the droplet exposure position allows the airflow and electric field to act cooperatively in a stable manner. In the voltage–air pressure pair, the key issue is whether the electrostatic stretching intensity is sufficient but not excessive, so that the aerodynamic effect can be amplified without destabilizing the jet. In terms of the force-balance model, the low-diameter region corresponds to a condition where the stretching index S is sufficiently high while the instability index I remains controlled.

Combined with the previous coupling-strength analysis and the localized SEM validation, the present response-surface results further confirm that parameter optimization in gas-assisted electrospinning should be understood as the identification of a multiphysics collaborative window, rather than the simple pursuit of a single-parameter extreme. Within the present experimental domain, the favorable process window can therefore be summarized as *V* ≈ 12 kV, $P_{air}$ ≈ 0.40–0.50 MPa, and *L_neelde_* ≈ 15 mm, where the balance among electrostatic stretching, aerodynamic assistance, and jet stability is most favorable for fiber refinement.

## 4. Conclusions

This study presents a comprehensive investigation into the intelligent optimization and multiphysics mechanism of gas-assisted electrospinning, integrating data-driven modeling, knowledge-guided algorithms, and physical theory.

First, regarding surrogate model construction, a systematic evaluation of kernel functions and sample sizes was conducted. The Matern 5/2 kernel with N = 30 samples was identified as the optimal configuration, providing a robust predictive basis while maintaining low experimental cost.

Second, the proposed BO+LLM collaborative framework demonstrated superior performance compared to both LLM-only and BO-only strategies. Crucially, the conventional RSM baseline predicted an unrealistically low optimum of 94 nm, whereas the experimentally validated diameter was 285 nm. In contrast, the BO+LLM strategy successfully located an experimentally verified minimum diameter of 239 nm. This highlights the superiority of adaptive, feedback-driven optimization over static polynomial models in navigating complex, nonlinear process spaces.

Third, through GPR-based parameter-response analysis and coupling-strength matrix evaluation, this work identified the critical coupled parameter pairs: *V*–$P_{air}$, $P_{air}$–$L_{needle}$, and *V*–$L_{needle}$. To interpret these couplings, a theoretical force-balance model was established, revealing that fiber refinement is governed by the competition between the stretching index *S* and the instability index *I*.

Finally, localized experiments and response-surface analysis validated that the favorable low-diameter region forms a multiphysics collaborative window centered around *V* ≈ 12 kV, $P_{air}$ ≈ 0.40–0.50 MPa, and $L_{needle}$ ≈ 15 mm. Within this window, electrostatic stretching and aerodynamic assistance are balanced to maximize *S* while suppressing *I*.

In summary, this work not only provides an efficient optimization pathway but also clarifies the specific physical mechanisms governing nanofiber refinement in gas-assisted electrospinning. The proposed strategy provides a practical pathway for the scalable and reproducible manufacturing of high-performance nanofiber-based electronic devices.

## Figures and Tables

**Table 1:** Experimental parameter ranges for the experiment.

Parameter	Symbol	Range	Unit	Description
Polymer concentration	*C*	10~16	wt%	Concentration range used to regulate solution viscosity and jet formation
Applied voltage	*V*	8~14	kV	Voltage applied between the needle and collector
Solution flow rate	*Q*	0~1.5	mL/h	Feeding rate of the precursor solution
Emitter–collector distance	*D*	9~18	cm	Distance between the needle tip and the collector
Air pressure	$P_{air}$	0~1.5	MPa	Auxiliary airflow pressure used to assist jet stretching
Needle extension	$L_{needle}$	10~20	mm	Needle protrusion length relative to the gas-assisted nozzle

**Table 2:** Predictive performance of different kernel functions under different sample sizes.

Total Sample Size (N)	Kernel	R^2^	RMSE (nm)	MAE (nm)	Estimated 95% CI (nm)
20	Matern 3/2	0.44	109	87	±214
20	Matern 5/2	0.54	118	77	±231
20	Squared Exponential	0.56	110	86	±216
30	Matern 3/2	0.65	98	57	±192
30	Matern 5/2	0.88	54	40	±106
30	Squared Exponential	0.78	78	54	±153
40	Matern 3/2	0.68	98	53	±192
40	Matern 5/2	0.84	64	39	±125
40	Squared Exponential	0.83	60	36	±118

**Table 3:** LOOCV robustness comparison of Matern 5/2 GPR models under different sample sizes.

Total Sample Size (N)	Kernel	LOOCV_R^2^	LOOCV_MAE (nm)	LOOCV_RMSE (nm)	Estimated 95% CI (nm)
30	Matern 5/2	0.83	35	55	±108
40	Matern 5/2	0.87	29	47	±92

**Table 4:** Quantitative comparison of iterative optimization performance among different strategies.

Strategy	Best Diameter (nm)	Best Run	Optimization-Stage Average Diameter (nm)	Low-Diameter Retention Rate (%)
LLM	289.69	12	312.39	0
BO	244.15	4	259.58	50
BO+LLM	239.47	4	251.01	90

## Data Availability

The original contributions presented in this study are included in the article. Further inquiries can be directed to the corresponding authors.
